# The Significance of the Sequence of Initiating and Promoting Actions in the Process of Skin Carcinogenesis in the Mouse

**DOI:** 10.1038/bjc.1955.23

**Published:** 1955-06

**Authors:** I. Berenblum, Nechama Haran


### THE SIGNIFICANCE OF THE SEQUENCE OF INITIATING AND PROMOTING ACTIONS IN THE PROCESS OF SKIN CARCINOGENESIS IN THE MOUSE.

I. BERENBLUM AND NECHAMA HARAN.

*From the Department of Experimental Biology, The Weizmann Institute of Science, Rehovoth, Israel.*

Received for publication February 3, 1955.

THE two-stage mechanism of carcinogenesis (see review: Berenblum, 1954*a*) provides the basis for a new approach to the problem of tumour pathogenesis. It postulates that a specific "initiating action" converts one or more normal cells into "dormant tumour cells", and that the latter require, for their evolution into visible tumours, a "promoting action", apparently involving a delay in maturation until a critical-size colony of dormant tumour cells is reached (Berenblum, 1954*b*). An essential feature of this system is that the respective mechanisms of the two stages must be different and independent, so that the completed process of carcinogenesis, which results when promoting action *follows* initiating action, should not occur when the procedure is reversed. [If carcinogenesis merely depended on a summation of two sub-optimal doses of (one strong and one very weak) carcinogen, or if it involved a summation of two actions possessing the same mechanism, e.g. two mutations, as implied in the theories of Fisher and Hollomon (1951), Arley and Iversen (1952), Nordling (1953) and others, then it should be immaterial which action came first.]

There is already some evidence in support of the dependence of carcinogenesis on a correct sequence of initiating and promoting actions—when croton resin (the active principle of croton oil) is applied to the skin for 26 weeks *before* a course of treatment with 3 : 4-benzpyrene, no speeding up of tumour production occurs, but when the croton resin is administered *after* a short course of benzpyrene treatment, carcinogenesis is greatly augmented (Berenblum, 1941*b*). Indeed, this observation was the starting-point of the studies on the promoting action of croton oil or resin (Berenblum, 1941*b*), which, in turn, provided a quantitative basis for the two-stage mechanism concept (Berenblum and Shubik, 1947, 1949).

However, to prove the point, it was necessary to carry out a more critical experiment—a comparison between tumour production resulting from a single application of a carcinogenic hydrocarbon (for initiating action) followed by 40 half-weekly applications of croton oil (for promoting action) and that occurring when the 40 half-weekly applications of croton oil are followed by a single application of the carcinogenic hydrocarbon. Since this had not previously been carried out, the present investigation was undertaken.

### METHODS.

The mice used for this investigation were females of the Swiss strain, previously pen bred, and since their acquisition in these laboratories, by brother-to-sister mating for 10–11 generations. They were fed on Purina Laboratory Chow (Rawlston Purina Co., U.S.A.) and water *ad libitum*, and kept in air-conditioned rooms at 21–22° C. At the start of the experiment, they were 3½ months old. The mice were entirely free from any known infection, and the mortality during the period of treatment (23 weeks) was negligible (2/42 deaths).

Before each application, the hair over an area of about 1 sq. cm., in the region of the shoulder blades, was carefully clipped with fine scissors. The solutions for application (1·5 per cent 9 : 10-dimethyl-1 : 2-benzanthracene or 5 per cent croton oil, both in liquid paraffin) were delivered by a glass rod. The resulting tumours were charted when first observed, and fortnightly thereafter. Papillomas that appeared in some cases on the ears were charted likewise.

### RESULTS.

In the first group, 22 mice received a single application of DMBA (1·5 per cent 9 : 10-dimethyl-1 : 2-benzanthracene in liquid paraffin) to the skin of the back; the mice were then left untreated for 3 weeks, and thereafter received twice-weekly applications of croton oil (5 per cent in liquid paraffin) for 20 weeks, and then kept for a further 15 weeks for observation. The results of the experiment are summarized in Table I.

TABLE I.—*Development of Tumours in Mice Treated with Dimethylbenzanthracene and Croton oil.*

| Treatment. | | Mouse No. | First appearance of tumour (weeks). | | Tumours. | | |
|---|---|---|---|---|---|---|---|
| Primary. | Secondary. | | (a). | (b). | Skin of back. | Ears. | Persistence. |
| DMBA × 1 | Croton oil × 40 | 28–1 | 10 | 7 | 2 P | — | + |
| | | | 14 | 11 | — | 2 P | + + |
| | | 28–2 | 10 | 7 | 2 P | — | + + |
| | | 28–3 | 10½ | 7½ | — | 6 P | + + |
| | | | 16½ | 13½ | 1 P | — | +/− |
| | | 28–4 | 11½ | 8½ | 4 P | — | + |
| | | 28–5 | 12 | 9 | 2 P | — | +/− |
| | | 28–6 | 12 | 9 | 2 P | — | +/++ |
| | | | 18 | 15 | — | 3 P | + + |
| | | 28–7 | 14 | 11 | 1 P | — | +/− |
| | | | 14 | 11 | — | 4 P | + + |
| | | 28–8 | 14 | 11 | 1 P | — | +/− |
| | | 28–9 | 14½ | 11½ | — | 1 P | +/− |
| | | 28–10 | 14½ | 11½ | 1 P | — | + + |
| | | 28–11 | 17½ | 14½ | — | 2 P | + + |
| | | 28–12 to 28–22 | — | — | — | — | — |
| | | Total | | | 16 P | 18 P | |
| Croton oil × 40 | DMBA × 1 | 29–1 | 16½ | — | 1 P | — | + + |
| | | 29–2 | 25 | 2 | 1 P | — | + |
| | | 29–3 to 29–20 | — | — | — | — | — |
| | | Total | | | 2 P | — | |

(a) = from commencement of primary treatment; (b) = from commencement of secondary treatment; P = papillomas. + = persisting without growth in size; + + = progressive growth in size; +/− = regressed after 6 weeks or more; +/++ = original tumour disappeared but others appeared later.

Eleven of the 22 mice developed papillomas which were, in most cases, multiple: in 5 mice the tumours were confined to the treated skin (10 papillomas); in 4 mice they developed both in the treated skin (6 papillomas) and on the ears (15 papillomas); and in 2 mice they arose only on the ears (3 papillomas). The latent period ranged from 7–14½ weeks from the start of the second treatment (croton oil), or 10–17½ weeks if calculated from the beginning of the experiment. No new tumours developed after that, though the croton oil treatment was continued for another 5 weeks and the animals observed for a further 15 weeks. (This is in keeping with the earlier results of Berenblum and Shubik, 1947.) The persistence, growth, or regression, of the tumours, are indicated in Table I. (The recording of a tumour as " regressed " denoted that it had at least persisted for 4 weeks, and papillomas that disappeared in less than 4 weeks were not counted.)

In the second group the procedure was reversed: 20 mice received twice weekly applications of croton oil; they were then left untreated for 3 weeks, and then given a single application of DMBA, the dosage and other conditions of the experiment being otherwise identical with those of Group 1. These mice were also kept for observation for 15 weeks after cessation of treatment.

The results of this second group (also summarised in Table I) were strikingly different from those of the first group: only two of the 20 mice developed tumours, in each case a solitary papilloma. One of these appeared in 16½ weeks from the commencement of the experiment, i.e. before the second treatment was even begun; the other after 25 weeks from the start of the experiment, i.e. 2 weeks after the second treatment. In the one case the tumour grew progressively; in the other it remained stationary. No papillomas appeared on the ears in this group.

None of the tumours of either group became malignant.

### DISCUSSION.

The results essentially confirm previous expectation on the basis of the two-stage mechanism of carcinogenesis. Tumours developed in large numbers when promoting action *followed* initiating action, but not when the procedure was reversed.

The two papillomas that developed in the second group can be readily discounted, since one of them arose even before the second treatment was given and the other only two weeks after the second treatment. Both must, therefore, be attributed to a weak carcinogen action of the croton oil itself, of which examples have been previously recorded in rare instances (Berenblum, 1941*a*; Salaman and Roe, 1953).*

Even if the second tumour were accepted, the result of this experiment would be: eleven out of 22 mice developed tumours when promoting action followed initiating action, while one out of 20 developed tumours when promoting action came first; or, if expresed in total number of tumours, 34 tumours developed in the first group and only 1 in the second.

### SUMMARY.

1. A single application of 9 : 10-dimethyl-1 : 2-benzanthracene to the mouse's skin, followed by 40 twice-weekly applications of croton oil, led to the develop-

* At the 6th International Cancer Congress, Sao Paulo, Rusch (1954) reported on a strain of mice in which skin papillomas developed in larger numbers after croton oil treatment.

ment of papillomas in half the treated animals. When the treatment was reversed, with the 40 applications of croton oil *preceding* the single application of the dimethylbenzanthracene, only 2 out of 20 mice developed papillomas, of which one appeared before the second treatment was begun.

2. These results confirm the principle on which the two-stage mechanism of carcinogenesis is based, namely, that the two stages—initiating and promoting—represent different and independent processes which do not act by summation, but only when administered in a particular sequence, whereby the initiating action converts some normal cells into dormant tumour cells while promoting action converts the dormant tumour cells into recognizable tumour masses.

### REFERENCES.

ARLEY, N. AND IVERSEN, S.—(1952) *Acta path. microbiol. scand.*, **31**, 164.

BERENBLUM, I.—(1941*a*) *Cancer Res.*, **1**, 44.—(1941*b*) *Ibid.*, **1**, 807.—(1954*a*) 'Advances in Cancer Research.' Acad. Press Inc., **2**, 129.—(1954*b*) *Cancer Res.*, **14**, 471.

*Idem* AND SHUBIK, P.—(1947) *Brit. J. Cancer*, **1**, 383.—(1949) *Ibid.*, **3**, 109.

FISHER, J. C. AND HOLLOMON, J. H.—(1951) *Cancer*, **4**, 916.

NORDLING, C. O.—(1953) *Brit. J. Cancer*, **7**, 68.

RUSCH, H. P.—(1954) Report at 6th International Cancer Congress, Sao Paulo.

SALAMAN, M. H. AND ROE, F. J. C.—(1953) *Brit. J. Cancer*, **7**, 472.


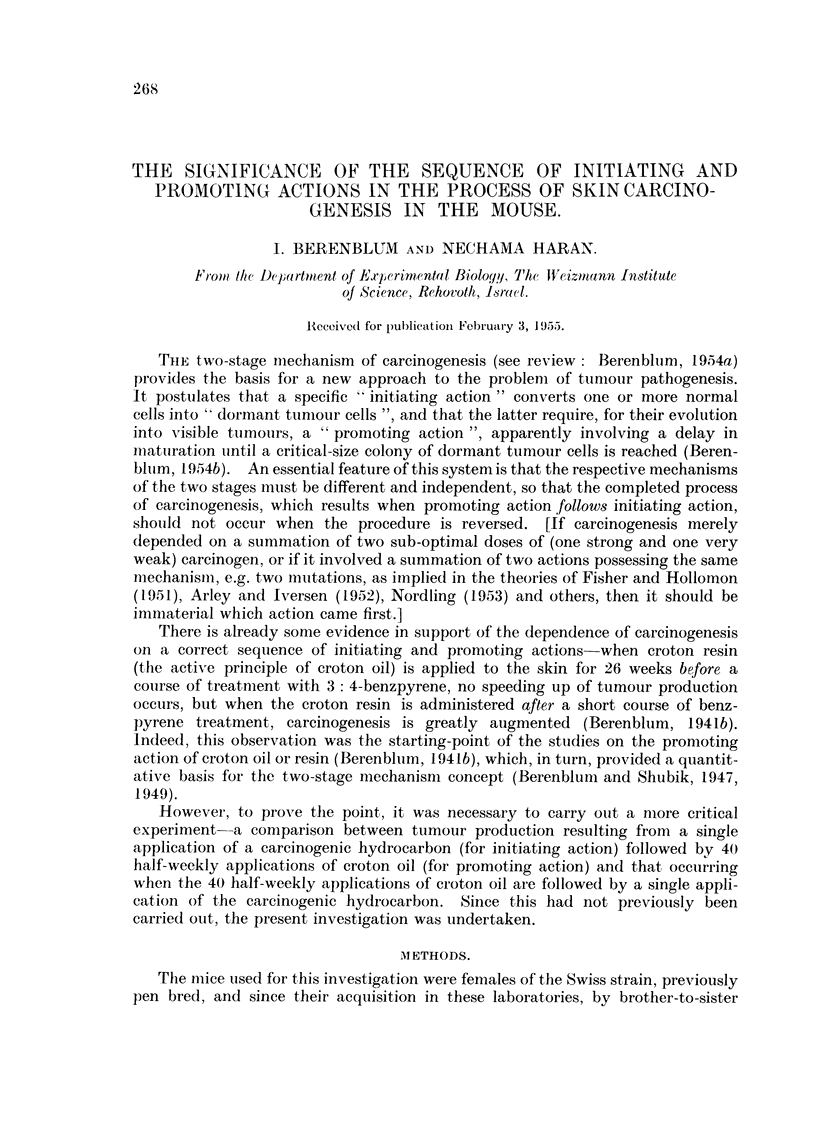

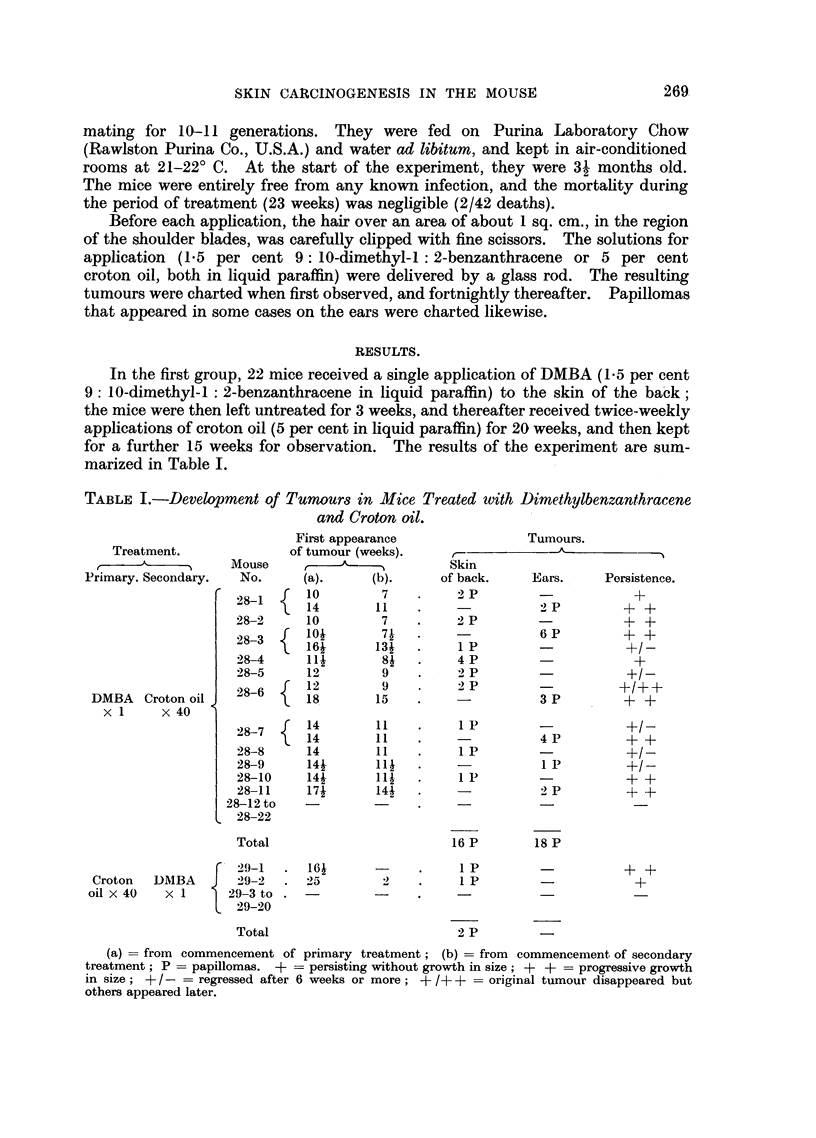

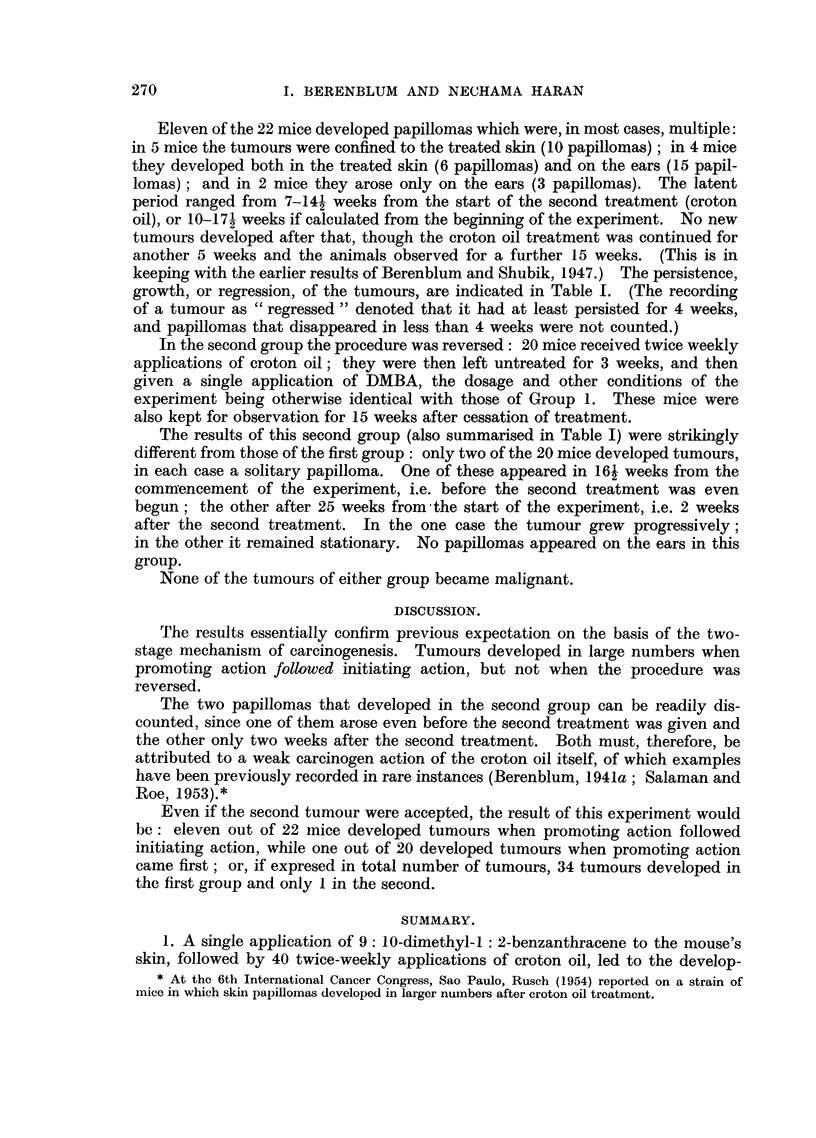

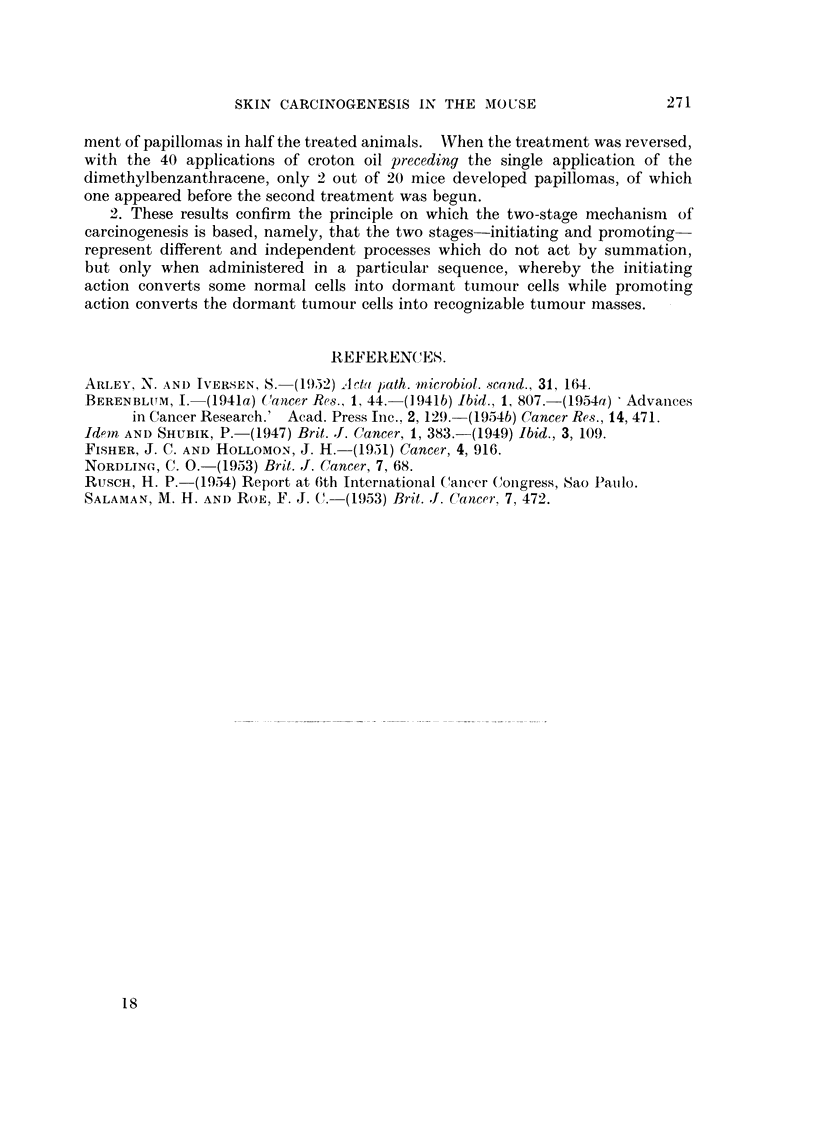


## References

[OCR_00213] ARLEY N., IVERSEN S. (1952). On the mechanism of experimental carcinogenesis. VI. Hit theoretical interpretation of some experiments of Berenblum & Shubik.. Acta Pathol Microbiol Scand.

[OCR_00219] FISHER J. C., HOLLOMON J. H. (1951). A hypothesis for the origin of cancer foci.. Cancer.

[OCR_00223] SALAMAN M. H., ROE F. J. (1953). Incomplete carcinogens: ethyl carbamate (urethane) as an initiator of skin tumour formation in the mouse.. Br J Cancer.

